# The Vitamin E Derivative Gamma Tocotrienol Promotes Anti-Tumor Effects in Acute Myeloid Leukemia Cell Lines

**DOI:** 10.3390/nu11112808

**Published:** 2019-11-17

**Authors:** Paola Ghanem, Annalise Zouein, Maya Mohamad, Mohammad H. Hodroj, Tony Haykal, Sonia Abou Najem, Hassan Y. Naim, Sandra Rizk

**Affiliations:** 1 Department of Natural Sciences, School of Arts and Science, Lebanese American University, Beirut 1102-2801, Lebanon; paola.ghanem@lau.edu (P.G.); annalise.zouein@lau.edu (A.Z.); maya.mohamed@lau.edu (M.M.); mohammadhassan.hodroj@lau.edu (M.H.H.); tony.haykal@lau.edu (T.H.); Sonia.abounajem@lau.edu.lb (S.A.N.); 2 Department of Physiological Chemistry, University of Veterinary Medicine Hannover, 30559 Hannover, Germany; hassan.naim@tiho-hannover.de

**Keywords:** leukemia, vitamin E, gamma tocotrienols, apoptosis

## Abstract

Acute myeloid leukemia (AML) is a blood cancer characterized by the formation of faulty defective myelogenous cells with morphological heterogeneity and cytogenic aberrations leading to a loss of their function. In an attempt to find an effective and safe AML treatment, vitamin E derivatives, including tocopherols were considered as potential anti-tumor compounds. Recently, other isoforms of vitamin E, namely tocotrienols have been proposed as potential potent anti-cancerous agents, displaying promising therapeutic effects in different cancer types. In this study we evaluated the anti-cancerous effects of γ-tocotrienol, on AML cell lines in vitro. For this purpose, AML cell lines incubated with γ-tocotrienol were examined for their viability, cell cycle status, apoptotic cell death, DNA fragmentation, production of reactive oxygen species and expression of proapoptotic proteins. Our results showed that γ-tocotrienol exhibits time and dose-dependent anti-proliferative, pro-apoptotic and antioxidant effects on U937 and KG-1 cell lines, through the upregulation of proteins involved in the intrinsic apoptotic pathway.

## 1. Introduction

Acute myeloid leukemia (AML) is a group of genetically heterogeneous neoplastic clonal disorders characterized by an uncontrolled increase in the number of immature de-differentiated malignant myeloblasts accumulating mostly in the blood, bone marrow and less often in other tissues [1]. If left untreated, AML can be fatal within few months after diagnosis as a result of opportunistic infections or bleeding.

Vitamins have shown great effectiveness in the treatment of several cancer types. Primarily, vitamins C, D and E appeared to play a significant role in improving the side effects of chemotherapy and radiation for lung, stomach, and prostate cancers [2]. Recently, members of the vitamin E family are being highly explored for cancer therapy purposes due to their various protective properties, medicinal benefits and low cytotoxicity [3].

The vitamin E family, composed by tocopherols and tocotrienols, is a group of plant derived lipid soluble compounds with antioxidant properties and well-reported protective effects against several diseases linked to oxidative damage including arthritis, Alzheimer’s disease, atherosclerosis, aging and cancer [4,5,6,7]. Tocopherols are most commonly found in dietary oil sources including vegetable oils, soybean, sesame and nuts, whereas, tocotrienols are primarily derived from palm oil, wheat, oat, barley, rice bran oil, rye and annatto (Bixa orellana) [4,8,9]. Four isoforms of tocopherols and that of tocotrienols, α, β, γ and δ, exist in nature, with saturated analogs belonging to tocopherols and unsaturated analogs comprising the tocotrienols class of vitamin E [10]. The previous focus of research on vitamin E included tocopherols due to their high bioavailability, however, recent findings revealed the abundant bioactivity of tocotrienols and reinforced their use as attractive avenues for basic and clinical research. Furthermore, several studies documented that tocotrienols possess profound anti-cancerous effects that are not elicited by tocopherols [4,11].

The gamma isomer of tocotrienols has been shown to exert profound anti-neoplastic activities in several cancer types. An in vitro study by Park et al. revealed that γ-tocotrienol enhances the expression of death receptor 5 (DR5), thus promoting apoptosis in breast cancer cells [12]. In addition, Zhang et al., demonstrated the dose-dependent anti-proliferative effects of γ-tocotrienol on the human colon cancer SW620 and HCT-8 cells [13]. In addition, Luk et al., proved that γ-tocotrienol downregulates the expressions of stem cell survival proteins, Id-1 and β-catenin, in prostate cancer cells [14]. Taking advantage of its antioxidant potential, γ-tocotrienols were also implicated in several combination therapies. For instance, its coupling to tyrosine kinase inhibitors such as gefitinib or erlotinib, resulted in the inhibition of epidermal growth factordependent mitogenic signaling in murine mammary tumor cells [15]. Similarly, Malaviya and Sylvester, showed, that a combined treatment involving γ-tocotrienol with PPARγγ antagonists such as GW9662 or T0070907, synergistically inhibited the cell growth of breast cancer cell lines, MCF-7 and MDA-MB-231 [16]. Those studies indicate that γ-tocotrienol alone, or in combination to other chemotherapeutic drugs, exert anti-cancerous effects on various cancer types. However, data on the efficacy of γ-tocotrienol in treatment of leukemia in general, and AML in specific, is still poorly studied. 

The present study aims to investigate the anti-cancerous effect of the vitamin E derivative, γ-tocotrienol, on human acute myeloid leukemia cells. Several experiments have been performed on U937 and KG-1 cell lines, to elucidate the efficacy of γ-tocotrienol, and unravel the underlying molecular mechanism. 

## 2. Materials and Methods

### 2.1. Cell Culture

Human AML cell lines U937 and KG-1 were obtained from ATCC and cultured in fresh Roswell Park Memorial Institute growth medium, RPMI1640, containing 25 mM Hepes and L-glutamine supplemented with 10% fetal bovine serum (FBS) and 100 U of penicillin and streptomycin, in a humidified incubator at 37 °C and 5% CO_2_.

### 2.2. Isolation and Culture of Mesenschnymal Stem Cells (MSCs) from Rat Bone Marrow

A single, 12-week old male Wistar rat weighing 180–220 g was provided by the animal facility at the Lebanese American University. The animal was maintained under optimal laboratory environment condition with ad libitium access to food and water. All experimental procedures were approved by the University’s Animal Care and Use Comity (ACUC) and complied with the Guide for the Care and Use of Laboratory Animals. MSCs were isolated from the rat’s bone marrow according to a modified procedure. Briefly, the rat was sacrificed by CO_2_ asphyxiation and both hind legs were aseptically removed. Femoral and tibial bones were then isolated and washed with 70% ethanol and placed in sterilephosphate buffered saline (Lonza) supplemented with 100 U/mL penicillin and 100 mg/mL streptomycin (Lonza). After removing the bone epiphyses, the bone marrows were flushed out using a needle filled with Dulbecco’s modified eagle medium (DMEM, Sigma-Aldrich) supplemented with 10% fetal bovine serum (GibcoTM) and 100 U/mL penicillin and 100 mg/mL streptomycin (Lonza). The cells collected were then incubated in vented flasks at 37 °C with 5% CO_2_. After five days of daily medium change, MSCs were identified by their spindle-shaped morphology as observed using the ZOE Fluorescent Cell Imager [17].

### 2.3. Drug Preparation

The vitamin E derivative, γ-tocotrienol stock solution was dissolved in dimethyl sulfoxide (DMSO), diluted in RPMI-1640, and used at various concentrations (10–50 µM). Etoposide, a topoisomerase II inhibitor with a proven efficacy on several cancer types including leukemia, was also prepared in DMSO, and used as a positive control at a concentration of 20 µM.

### 2.4. Cell Viability Assay

AML cells were seeded in triplicate wells in flat-bottomed 96-well plates at a density of 3 × 10^5^ cells/mL (final volume of 100 µL) and incubated overnight. Γ-tocotrienol was added onto the cells and incubated for 24 or 48 h. At each time point, cell viability was evaluated using an MTS (3-(4,5-Dimethylthiazol-2-yl)-5-(3-carboxymethoxyphenyl)-2-(4-sulfophenyl)-2H-tetrazolium salt) assay kit (Promega) according to the manufacturer’s instructions. MTS reagent was added to the wells and optical density (OD) values of absorbance were measured at 490 nm using a Varioskan Flash plate reader (Thermo Fisher Scientific). The percentage of proliferating cells was determined relative to 100 percent viability of control untreated cells.

### 2.5. Cell Cycle Analysis

Cells were seeded in 24-well plates at a density of 3 × 10^5^ cells/mL (final volume of 700 µL), and treated with increasing concentrations of γ -tocotrienol (10–50 µM). After 24 h of the treatment, cells were harvested, washed with 1X PBS, fixed using 70% cold ethanol, treated with 100 µg/mL ribonuclease I, RNase, followed by propidium iodide (PI) staining. The cell’s DNA content was assessed and quantified by flow cytometry using C6 flow software. Ten thousand gated events were recorded per sample to assess the proportions of cells in different stages of the cell cycle. The cell cycle analysis was based on the estimation of the frequency of cells in each phase of the cell cycle depending on the cells’ DNA content, in which sub-G0/G1 (pre-G1) cells are <2n, G0/G1 cells are 2n, S cells are >2n but <4n, whereas M phase cells were 4n.

### 2.6. Apoptosis Detection

Cells were plated in 24 well-plates at a density of 3 × 10^5^ cells/mL and incubated for 24 h. After treatment with γ-tocotrienol, cells were collected and stained with 5 µL PI and 5 µL annexin V-FITC, (An), for 15 min at 37 °C in the dark and then analyzed using flow cytometer (annexin V-FITC Apoptosis Staining Kit, Abcam Inc., Cambridge, UK) using the florescent dye FITC signal detector and the phycoerythrin emission signal detector for annexin V-FITC binding and PI staining, respectively. Obtained data was analyzed using BD Accuri C6 Plus Software. An^−^/PI^−^ cells were viable cells. An^+^/PI^−^ and An^+^/PI^+^ cells were cells in their early and late apoptotic stage respectively. Total apoptosis was the sum of early and late apoptotic cells. 

### 2.7. DNA Fragemnetation using Cell Death ELISA

Cells were plated in 6-well plates at a density of 3 × 10^5^ cells/mL (final volume of 2000 µL). Following treatment for 24 h, DNA fragmentation was assayed using cell death detection ELISA kit (Roche Applied Science, Mannheim, Germany) according to the manufacturer’s instructions. Briefly cytosolic DNA was extracted, placed in microtiter plates coated with anti-histone antibodies, followed by the addition of peroxidase-conjugated anti-DNA antibodies. After incubation, 2,2′-azino-di [3-ethylbenzthiazolin-sulfonate] (ABTS) was added and DNA fragmentation was measured using the Varioskan Flash plate reader at 405 nm. Enrichment factor (EF) was calculated as the recorded absorbance of each sample, divided by that of the untreated control cells. 

### 2.8. Western Blotting

Total proteins were extracted from U937 cells following 24 h of treatment using the Qproteome mammalian protein preparation kit (Qiagen Co., Hilden, Germany). “DC Protein Assay II” kit (Bio-Rad, Hercules, CA, USA) was then used to determine protein concentrations. 

Western blot assays were then performed to measure the protein expression of Bax, Bcl-2, caspase-3, cytochrome c and cleaved PARP-1. Sodium dodecyl sulfate-polyacrylamide gel electrophoresis (SDS-PAGE) was carried out under standard conditions to separate proteins, which were then transferred onto polyvinylidene difluoride (PVDF) membranes at 0.25 mA for 90 min. The membranes were then blocked using 5% skimmed dry milk in PBS containing 0.05% Tween-20 for an hour on the shaker at 40 rpm. Each membrane was then incubated with the specific primary antibody at a concentration of 1:500 overnight at 4 °C for 90 min. The membranes were then washed for 30 min using 1X PBS containing Tween-20 then incubated with the specific secondary antibody at a concentration of 1:2500. An hour later, the membranes were washed again and the bands were visualized on the ChemiDoc XRS+ machine (Bio-Rad, Hercules, CA, US), using western blotting chemiluminescent reagent enhanced chemiluminescence (ECL) (GE Healthcare, Chicago, IL, USA). The levels of protein expression were later quantified and compared using the ImageJ software.

### 2.9. Detecteion of Reacrtive Oxygen Species 

KG-1 cells were incubated with the cell permeant fluorogenic dye 2′,7′–dichlorofluorescin diacetate (DCFDA) for 30 min (Abcam, Cambridge, UK). DCFDA measures hydroxyl, peroxyl and other reactive oxygen species (ROS) activity within the cell. The cell suspension was then centrifuged at 1500 rpm for 5 min and the supernatant containing the stain solution was discarded and the cell pellet was then re-suspended in an FBS-containing supplemental buffer. Afterwards, cells were plated in triplicates in 96-well dark plates, and then directly treated with different concentrations of γ-tocotrienol alone and γ-tocotrienol along with 75 μM Tert-Butyl hydrogen peroxide (TBHP), which was used as a potent ROS inducer. Following 2 h of incubation at 37 °C, fluorescent 2′,7′-dichlorofluorescein (DCF) was quantified by fluorescent spectroscopy on the Varioskan™ LUX multimode microplate reader (Thermo Fisher Scientific, Waltham, MA, USA).

### 2.10. Statistical Analysis

All the experiments were carried out in triplicates and each experiment was repeated three times. Data are reported as mean ± SEM. Statistical analysis was done using two-way analysis of variance (ANOVA). The level of significance (*p*-values) upon comparing control versus treatment was set at *p* < 0.05.

## 3. Results

### 3.1. Effect of γ-Tocotrienol on the Proliferation of AML Cell Lines

**Figure 1:**
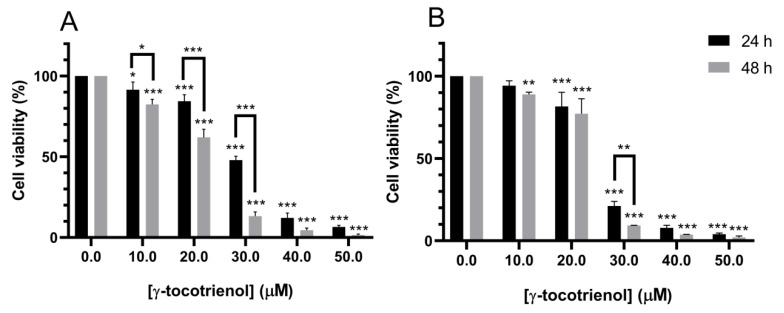
Effect of γ-tocotrienol on the cell viability of U937 (**A**) and KG-1 (**B**) cell lines. U937 and KG-1 were treated with various concentrations of γ-tocotrienol (0–50 µM) for 24 and 48 h. Cell viability was examined using MTS assay. *, ** and *** indicate *p* < 0.05, *p*  <  0.001 and *p* <  0.0001 respectively.

Treatment with increasing doses of γ-tocotrienol for 24 h reduced the proliferation of U937 and KG-1 cells in a dose-dependent manner with a half inhibitory concentration (IC50) of 29.43 and 25.23 µM, respectively. γ-tocotrienol also induced a dose and time-dependent decrease in the proliferation of both cell lines after 48 h of treatment with IC50s of 22.47 and 24.01 µM for U937 and KG-1 cells respectively (Figure 1).

### 3.2. Effect of γ-Tocotrienol on the Proliferation of Mesenchymal Stem Cells

**Figure 2:**
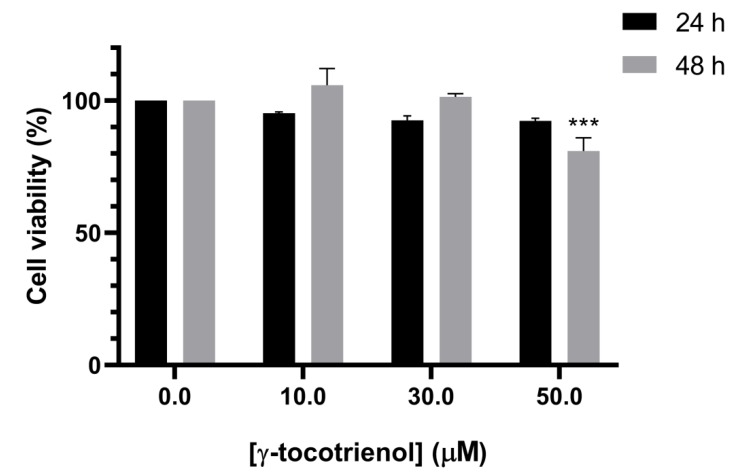
Effect of γ-tocotrienol on the cell viability of normal mesenchymal stem cells. MCS cells incubated with various concentrations of γ-tocotrienol (10, 30 and 50 µM) for 24 and 48 h and the cell viabilities were examined using an MTS assay kit. *** indicates *p* <  0.0001.

To test the selectivity of the elicited growth inhibitory effects of γ -tocotrienol against cancer cells, mesenchymal stem cells (MSCs) were treated with the various concentrations of γ-tocotrienol for 24 and 48 h. Cell viability was then examined by MTS reagent. As shown in Figure 2, the cell viability of MSCs was not significantly altered upon γ-tocotrienol treatment, as compared to control untreated MSCs, except with the highest concentration, 50 µM, after 48 h. This indicates that γ-tocotrienol can cause cell death in leukemic cell lines with minor effects on normal human cells (Figure 2). All remaining experiments were therefor performed with 24 h exposure, which revealed no cytotoxic effects on normal MSCs. 

### 3.3. Effect of γ-Tocotrienol on the Cell Cycle Progression of AML Cell Lines

**Figure 3:**
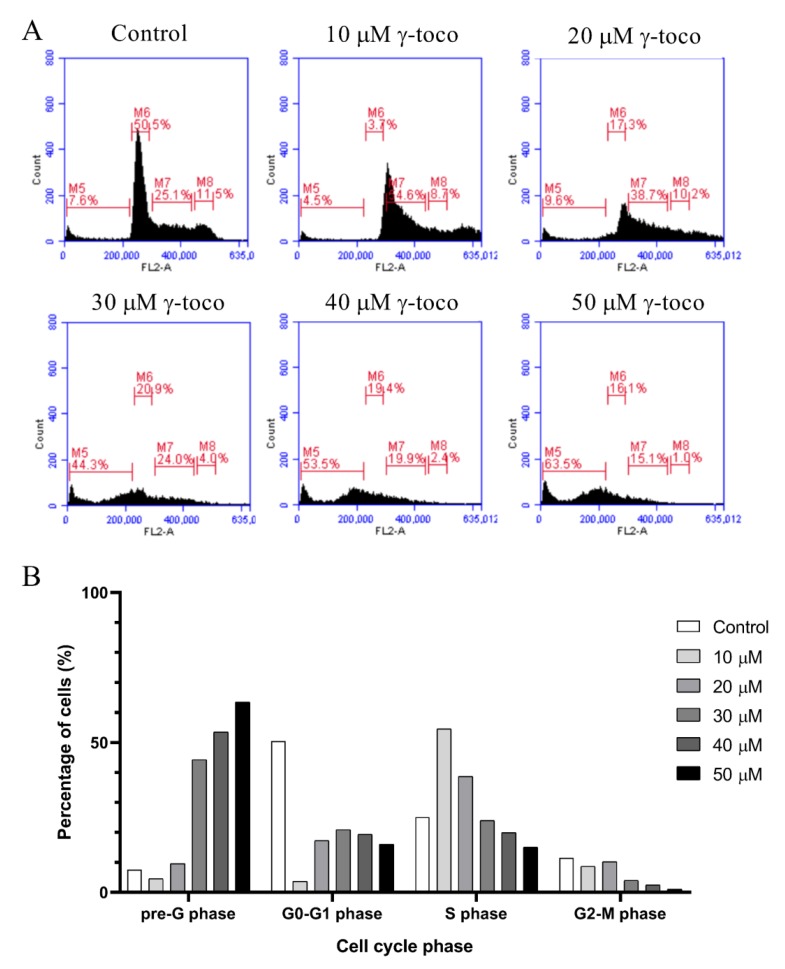
Effect of γ-tocotrienol on the cell cycle progression of U937. (**A**) Propidium iodide staining and flow cytometric analysis of cell cycle distribution of U937 cells treated with γ-tocotrienol for 24 h. The percentage of each cycle was determined using C Flow software. M5: sub-G1, M6: G0-G1 phase, M7: S phase, M8: G2/M phase. (**B**) Histogram analysis showing the percentage of cell cycle distribution of U937 cells treated with γ-Tocotrienol.

**Figure 4:**
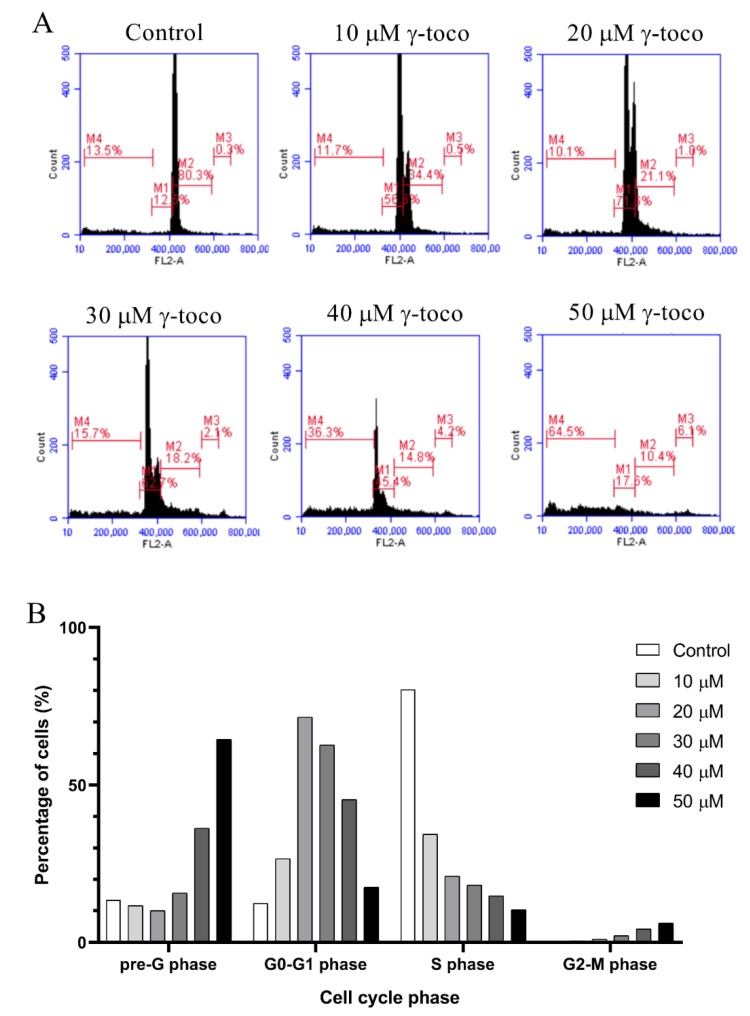
Effect of γ-tocotrienol on the cell cycle progression of KG-1 cell line. (**A**) Propidium iodide staining and flow cytometric analysis of cell cycle distribution of KG-1 cells treated with γ-tocotrienol for 24 h. The percentage of each cycle was determined using C Flow software M5: sub-G1, M6: G0-G1 phase, M7: S phase, M8: G2/M phase. (**B**) Histogram analysis showing the percentage of cell cycle distribution of KG-1 cells treated with γ-tocotrienol.

The flow cytometric cell cycle analysis of control untreated U937 cells showed accumulation of the cells in the G0/G1 phase. Treated cells, however, showed a dose-dependent increase in the percentage of dead cells in the sub-G0/G1 phase of the cell cycle, reaching 63.5% with 50 µM dose of γ-tocotrienol (Figure 3). Similarly, the flow cytometric cell cycle analyses of KG-1 cells treated with γ-tocotrienol showed a dose-dependent increase in the percentage dead cells at the sub-G0/G1 phase, to be 64.5% with 50 µM γ-tocotrienol (Figure 4).

### 3.4. Effect of γ-Tocotrienol on Apoptosis in AML Cell Lines

**Figure 5:**
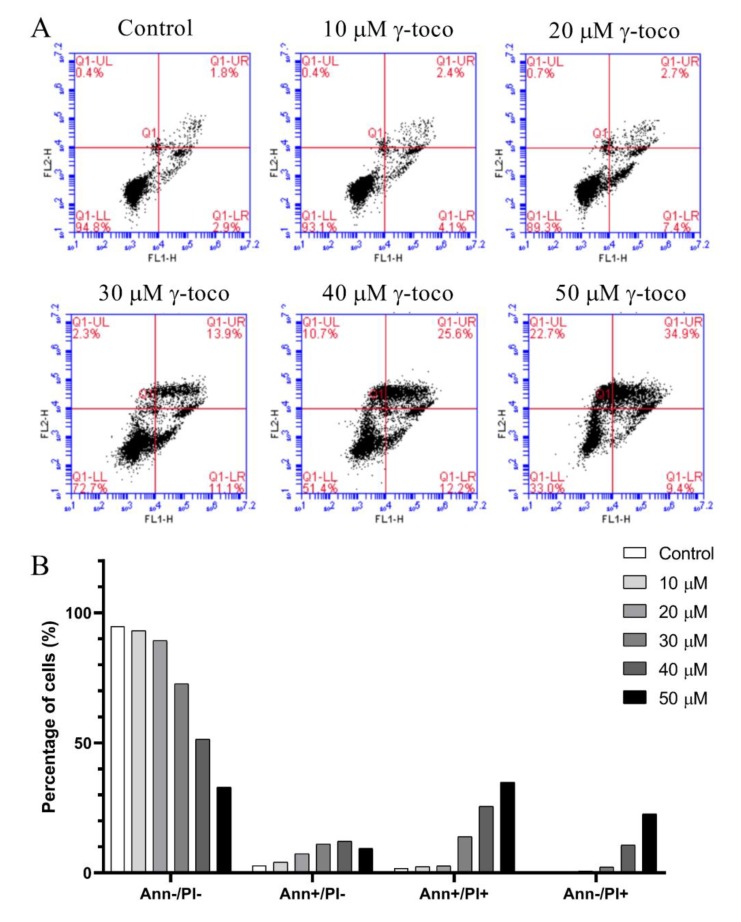
Effect of γ-tocotrienol on cell death in U937 cells. (**A**) Apoptosis of U937 cells was measured by flow cytometry following dual staining with annexin V-FITC (FL1-H) and propidium iodide (FL2-H). Cells were treated with γ-tocotrienol 24 h. Cells were then analyzed using the C Flow software: the lower left quadrant shows cells which are negative for both propidium iodide (PI) and annexin (normal cells). The upper left quadrant shows only PI positive cells (necrotic). The lower right quadrant shows annexin positive cells (early apoptotic). The upper right quadrant shows annexin and PI positive cells (late apoptotic cells). (**B**) Histogram analysis showing the percentage of cells in each quadrant before and after treatment with γ-tocotrienol.

**Figure 6:**
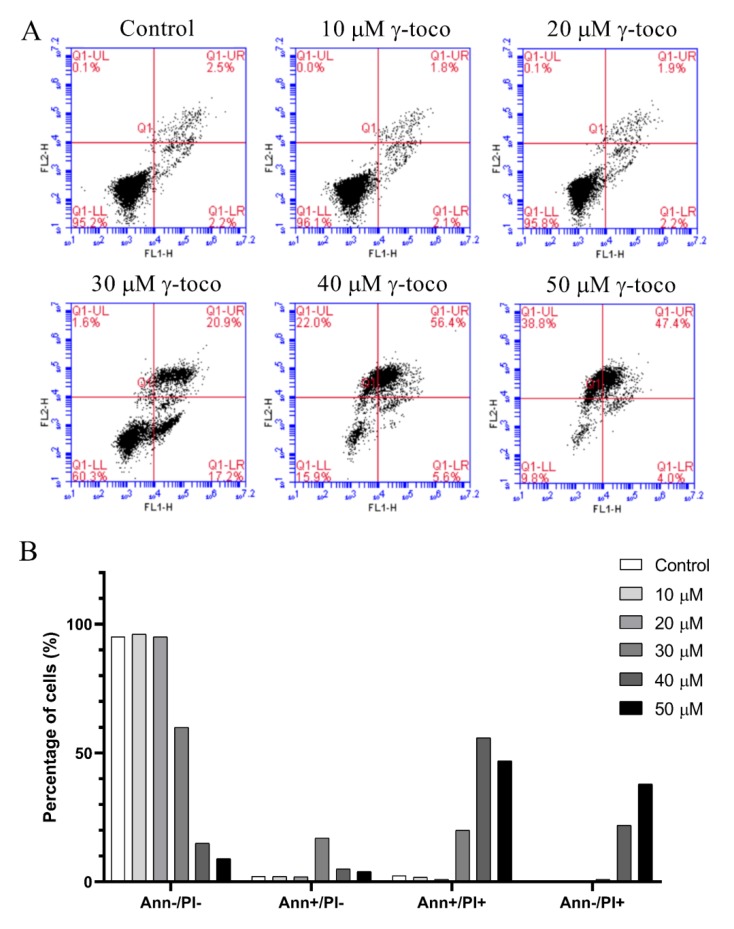
Effect of γ-tocotrienol on cell death of KG-1 cells. (**A**) Apoptosis of KG-1 cells was measured by flow cytometry following dual staining with annexin V-FITC (FL1-H) and propidium iodide (FL2-H). Cells were treated with γ-tocotrienol for 24 h. Cells were then analyzed using the C Flow software. (**B**) Histogram analysis showing the percentage of cells in each quadrant before and after treatment with γ-tocotrienol.

The annexin V/propidium iodide apoptosis staining assay was performed to assess cell death and detect whether the type of cell death induced by γ-tocotrienol in U937 and KG-1 cell lines, was apoptotic, necrotic, or both, The annexin V/PI flow cytometric analysis of U937 cells showed a decrease in the viable population (annexin V^−^/PI^−^) with increasing concentrations of γ-tocotrienol reaching 33% with the highest dose of 50 µM after 24 h. In parallel to this decrease, the percentage of cells in the late apoptotic stage (annexin V^+^/PI^+^) increased in a dose-dependent manner, reaching 34.9% with 50 µM γ-tocotrienol. The population of cells in the early apoptotic stage (annexin V^−^/PI^+^) also showed a slight increase (Figure 5). The flow cytometric analysis of KG-1 cells was similar to the results obtained in U937 cells. The viability decreased in treated cells with increasing doses of γ-tocotrienol. However, the population of cells in the late apoptotic (annexin V^+^/PI^+^) and early apoptotic cells (annexin V^−^/PI^+^) appeared to be higher than those obtained for U937, reaching 56.4% and 38.8%, respectively (Figure 6). 

### 3.5. Effect of γ-Tocotrienol on DNA Fragmentation in AML Cell Lines

**Figure 7:**
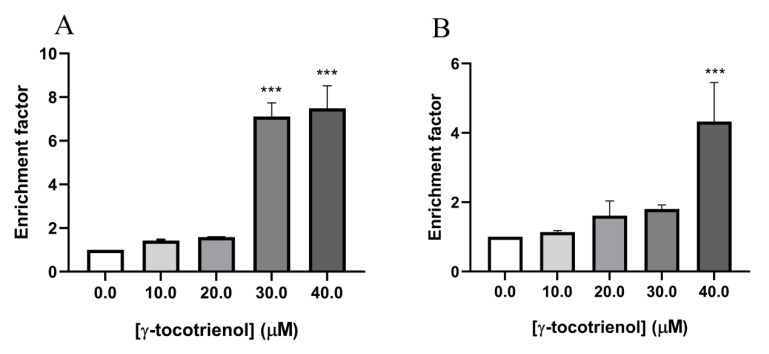
Effect of γ-tocotrienol on DNA fragmentation in U937 (**A**) and KG-1 **(B**) cell lines. Results show a dose-dependent increase in apoptotic enrichment factor upon treatment with γ-tocotrienol. Data represent mean ± standard error of the mean (SEM) from three independent experiments. *** indicates a significantly different mean values compared with control cells with *p* < 0.0001.

To further confirm the effect of γ-tocotrienol on apoptosis in the two AML cell lines, U937 and KG-1 were treated with γ-tocotrienol for 24 h, then sandwich ELISA was performed in order to quantify cytoplasmic histone-associated DNA fragments, a hallmark of apoptosis, in response to treatment with increasing concentrations of γ-tocotrienol (10–40 µM). The absorbance measurements recorded which reflected the amounts of apoptotic histone/DNA were further calculated on a scale of apoptotic enrichment factor based on the manufacturer’s instructions. The results obtained reveal an increase in DNA fragmentation in U937 cells with increasing concentrations of γ-tocotrienol reaching a seven-fold increase at 40 µM γ-tocotrienol (Figure 7A). Similarly, results show an approximately 4.5-fold increase in the apoptotic enrichment factor upon treating KG-1 cells with 40 µM of γ-tocotrienol (Figure 7B).

### 3.6. Effect of γ-Tocotrienol on the Expression of Proteins involved in Pro-Apoptotic and Anti-Proliferative Pathways

**Figure 8:**
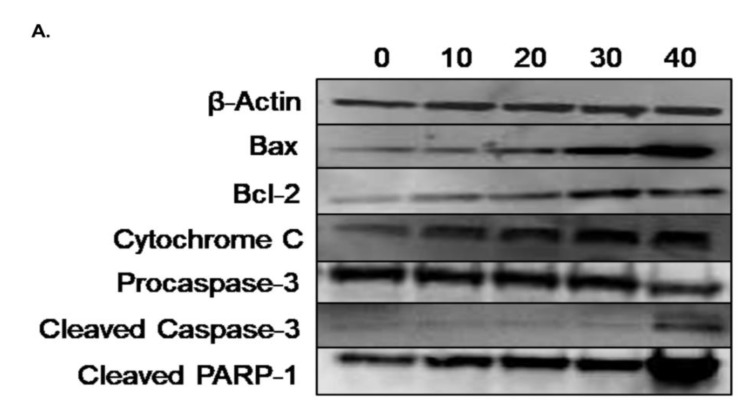

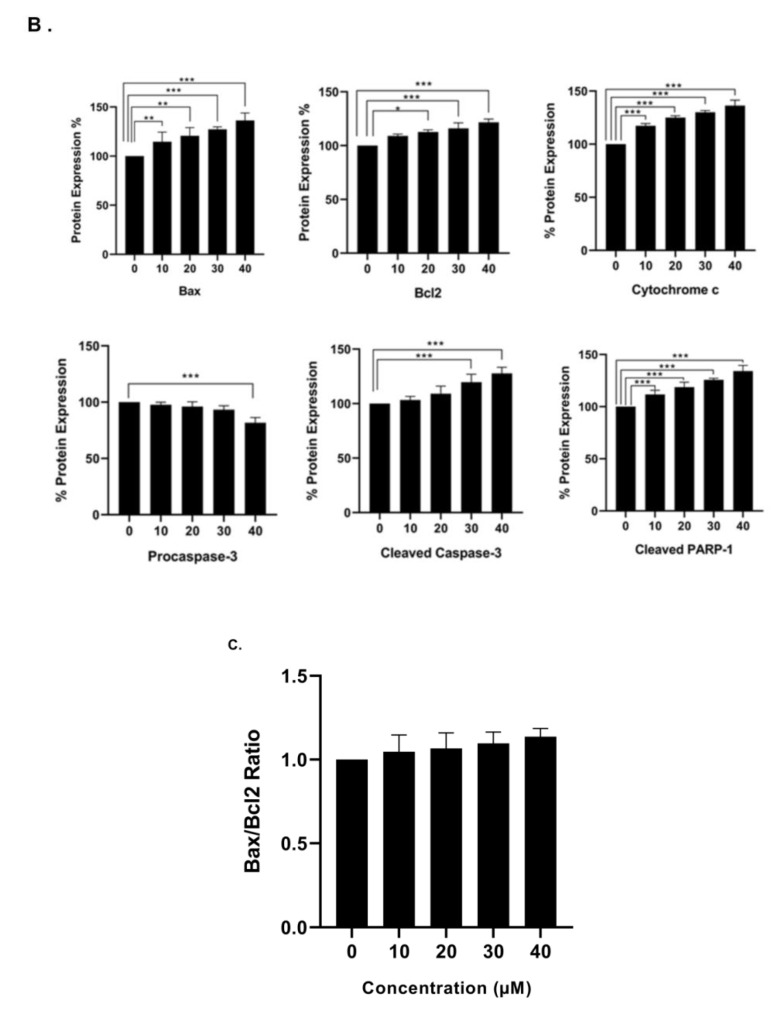
Western blot analysis showing the effect of γ-tocotrienol on the expression levels of apoptosis related proteins in U937 cell line. (**A**) Representative western blotting images for the expression of Bax, Bcl-2, caspase-3, cytochrome c, and cleaved PARP-1 in U937 cells following treatment with γ-tocotrienol for 24 h. (**B**) Quantification analysis of the western blots. (**C**) Quantification of Bax to Bcl2 ration. Values represent the percent expression relative to control, normalized to β-actin, for a total of three western blots. *, ** and *** indicate *p* < 0.05, *p*  <  0.001 and *p* <  0.0001 respectively.

In order to examine the molecular mechanism through which γ-tocotrienol exerts its anti-proliferative effects, western immunoblotting assays were performed to assess the expression of various apoptosis related proteins using Beta-actin as a house-keeping protein. The expression of the pro-apoptotic protein Bax was significantly upregulated by tocotrienol treatment in a dose-dependent manner as compared to control expression, whereas the expression of the anti-apoptotic protein Bcl-2 was slightly but significantly increased upon tocotrienol treatment; the Bax/Bcl-2 ratio showed an increase in favor of apoptotic cell death rather than cell survival. Tocotrienol at 40 µM significantly downregulated the expression of pro-caspase-3 and it also increased the expression of the active cleaved form. The pro-apoptotic effect of γ-tocotrienol was also assessed by measuring the expression of cytochrome c and cleaved PARP-1, both of which were significantly up-regulated in a dose-dependent manner, reaching 36% and 34% increase in protein expression, respectively, with the highest dose of 40 µM γ-tocotrienol (Figure 8).

### 3.7. Effect of γ-Tocotrienol on ROS Production in KG-1 Cell Line

**Figure 9:**
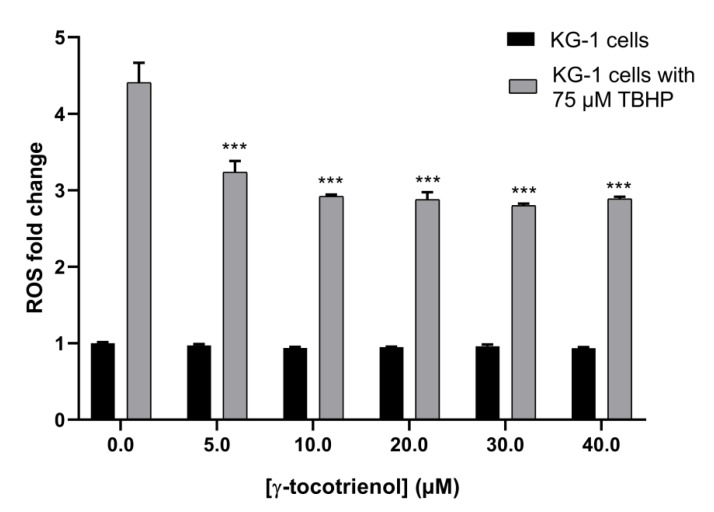
Effect of γ-tocotrienol on ROS level in KG-1 cells. TBHP was used in combination with γ-tocotrienol as a positive control in order to magnify the scale of change. γ-tocotrienol decreased ROS production in KG-1 cells in a dose-dependent manner. Data represent mean ± SEM from three independent experiments. *** indicates *p* <  0.0001.

The ROS assay was performed to assess the effect of γ-tocotrienol on the oxidative stress in AML cells. The assay relies on measuring the amount of produced reactive oxygen species in the AML cell line, KG-1, using TBHP as a potent ROS-inducer. Results showed no significant change in ROS production in cells treated with increasing doses of γ-tocotrienol indicating that its anti-cancerous activity is ROS-independent. Interestingly, an approximate 1.5-fold decrease in the level of cellular ROS production upon tocotrienol treatment was observed upon inducing ROS with TBHP, suggesting an antioxidant activity of γ-tocotrienol (Figure 9).

## 4. Discussion

Several *in vivo* and in vitro studies have demonstrated that the vitamin E derivative, γ-tocotrienol, has valuable anti-cancerous effects on various cancer types including breast, colon, prostate, pancreatic and lung cancers [3,6,14]. However, no studies have investigated the effect of γ-tocotrienol on acute myeloid leukemia. Thus, in the present study we aimed to investigate the anti-tumor effects of γ-tocotrienol on human AML cell lines, KG-1 and U937, in vitro. All results obtained were in accordance with previously published studies of γ-tocotrienol on other cancer types, whether given alone, pre-applied, or in synergistic combination to chemotherapeutic drugs. 

The anti-proliferative potential of γ-tocotrienol on U937 and KG-1 cells after 24 and 48 h of treatment, was highly indicative as the percent viability of both cell lines decreased in a dose and time-dependent manner upon treatment γ-tocotrienol. Similar to our findings, Ng KL et al. in 2016, demonstrated that γ-tocotrienol induced a dose-dependent proliferation inhibitory effect on the chronic myeloid leukemia cell line, K562, after 24 h of treatment with an IC50 of 30.962 µM, which is approximately similar to the IC_50_s obtained in our study at 24 h of treatment with γ-tocotrienol, 29.43 and 25.23 µM for U937 and KG-1 cells respectively [3]. Furthermore, Wei-Li Xu and his colleagues showed that γ-tocotrienol inhibited the proliferation of the human colon carcinoma HT-29 cells for 48 h of treatment with an IC_50_ of 31.7 µM, greater than the IC_50_s detected in our study at 48 h treatment: 22.47 and 24.01 µM for U937 and KG-1 respectively [18].

To test the selectivity of cytotoxicity of γ-tocotrienol on AML cells, cell viability of normal stem cells exposed γ-tocotrienol was measured. We obtained no significant change in cell viability, indicating that the potent cytotoxicity of γ-tocotrienol does not extend towards normal cells. Our results are in accordance with the results obtained by Yap et al. showing that treatment of the prostate cancer cell line PC-3 and normal prostate epithelial cells, PZ-HPV-7, with increasing dosage of γ-tocotrienol suppressed the proliferation of PC-3 without affecting the proliferation rate of PZ-HPV-7 [19].

The flow cytometric cell cycle analysis conducted in this study showed that γ-tocotrienol induces a shift in the cell cycle of treated cells, as compared to the controls, towards the sub-G0/G1 phase indicating the induction of cell death. Similar to our findings, Yap and his colleagues showed that γ-tocotrienol induced a dose-dependent elevation in the population at pre-G1 phase of the cell cycle of the treated prostate cancer cell lines LNCaP and PC-3 compared to the control untreated cells [19]. 

In order to verify that γ-tocotrienol induces cell death as assumed through apoptosis rather than necrosis, we further performed the flow cytometric annexin V-FITC/PI apoptosis detection assay followed by the cell death detection ELISA. Results of the annexin V/PI assay indicated that γ-tocotrienol induces apoptotic cell death in AML cells, as the percentage of cells in the late apoptosis quadrant (annexin V+/PI+) increased upon treatment for both cell lines, U937 and KG-1. In a similar manner, Bachawal et al. concluded that γ-tocotrienol in combination to erlotinib or gefitinib but not trastuzumab, induces apoptosis in +SA mammary tumor cells, as annexin V staining demonstrated an increase in the percentage of cells in the annexin V^+^/PI^−^ quadrant indicating that treated cells are in the early apoptosis stage [20]. The apoptotic effect was then confirmed by cell death detection ELISA as γ-tocotrienol appeared to be inducing a dose-dependent DNA fragmentation in AML cells, which is a hallmark of apoptosis. Similarly, Sakai et al. proved the apoptotic potential of γ-tocotrienol, through assessing its effect on DNA fragmentation in liver rat hepatoma dRLh-84 cells [21]. Wilankar and colleagues reported that γ-tocotrienol plays a role in inducing apoptosis via multiple apoptotic signaling pathways, including the death receptor pathway (extrinsic), the mitochondrial pathway (intrinsic), or other mechanisms like the endoplasmic reticulum (ER)-mediated apoptotic pathway [22]. Rickmann et al. and Sun et al. indicated in their studies on pancreatic and stomach cancers respectively, that γ-tocotrienols’ apoptotic activity is mainly via the mitochondrial intrinsic pathway [23,24]. Their outcome was compatible with ours, as the western blots analysis of our studied proteins, also suggested that γ-tocotrienol induces its apoptotic activity via the intrinsic pathway. γ-tocotrienol treatment resulted in the upregulation of the expression of the pro-apoptotic protein Bax in U937 cells. The anti-apoptotic protein Bcl-2 also appears to be increasing in treated cells. However, the Bax/Bcl-2 ratio was increased, thus the net effect of γ-tocotrienol was in favor of apoptotic cell death rather than cell survival. Furthermore, γ-tocotrienol was proven to induce apoptosis in AML cells through the activation of caspase-3, release of cytochrome c, and cleavage of PARP-1, as their expressions at the protein level appeared to increase in a dose-dependent manner. Our results were also in accordance with the results obtained by Ng KL et al., in which they observed a significant upregulation in the expression of various pro-apoptotic proteins belonging to the Bcl-2 family, including Bax, in addition to an enhanced expression of the pro-survival protein Bcl-2 in K562 CML cells treated with γ-tocotrienol [3]. In addition, Yap at al. reported the activation of PARP and procaspases (3, 7, 8, and 9) in the prostate cancer cell line, PC-3, as their cleaved products increased upon treatment with different concentrations of γ-tocotrienol. In their study, unlikely with what was observed in our current study, γ-tocotrienol didn’t seem to exert an effect on the expression of Bax, yet the expression of Bcl-2 was significantly downregulated, leading to a similar end-result yielding a higher Bax/Bcl-2 ratio, thus favoring apoptosis in PC-3 cells [19]. After demonstrating the anti-cancerous effects of γ-tocotrienol, through the induction of cell death in AML cells via apoptotic intrinsic signaling pathways, we moved on to test its effect on the oxidative state in AML cells. The ROS assay performed shows that γ-tocotrienol alone did not affect ROS production in cells, indicating that its anti-cancerous activity is not ROS-mediated. However, γ-tocotrienol in combination with TBHP suppresses the production of ROS in AML cells in a dose-dependent manner, suggesting that γ-tocotrienol has an antioxidant potential. Abd Hafid and Iran focused in their study on explaining the antioxidant activity of γ-tocotrienol. They showed that various vitamin E forms, especially γ-tocotrienol, when used in combination with chemotherapeutic drugs in treatment of AML, exhibit antioxidant activity which aids in reducing the oxidative stress generated as a drawback of chemotherapy, thus reducing the side effects of the treatment and achieving better survival outcomes [25].

## 5. Conclusions

Treatment of the AML cell lines, U937 and KG-1 with γ-tocotrienol led to a significant decrease in their viability, an increase in DNA fragmentation accompanied by an increase in the number of cells at the late apoptotic stage, indicating a profound apoptotic response to treatment. The induction of apoptotic death was featured by the elevated expression of cytochrome C, cleaved PARP and cleaved caspase 3 in addition to the increase in Bax/Bcl-2 ratio indicating the activation of the intrinsic apoptotic pathway. Our results indicate that γ-tocotrienol is an effective cytotoxic and anti-proliferative agent for AML treatment. This study also supports the potential antioxidant beneficial properties of γ-tocotrienol, which can be used for an effective improvement in cancer treatment. Future in vivo and in vitro studies are needed to further examine the efficacy of γ-tocotrienol in the treatment of AML.
